# Maternity and Mothering: The Influence of the Insurance Act upon the Practice of Midwifery

**Published:** 1914-07-18

**Authors:** 


### MATERNITY AND MOTHERING.

### The Influence of the Insurance Act upon the Practice of Midwifery.

When it became known that a maternity benefit, in the shape of a grant of thirty shillings per birth to insured women and the wives of insured men, was to be part of the Insurance Act, there was much speculation as to the possibility of sweeping alterations in the obstetrical conditions of the insured classes. Some looked for a much larger proportion of medically supervised confinements, some to an improvement in the emoluments and so in the status and proficiency of the trained midwife, some to the speedy extinction of the dirty, dangerous Gamp. The avowed object of the maternity benefit was to improve the whole of the environment of the new-made mother; and many ardent social reformers regarded material creature comforts as more-to-be-desired results of the thirty shillings benefit than an improvement in the general level of obstetric practice among the employed classes.

It is, naturally, an open question whether the health, comfort, and peace of mind of mothers of the labouring class are most likely to be promoted by better food, or by better hygienic management, or by better skill in midwifery. Everyone will agree that all these conditions need improving, and that thirty shillings per case will not suffice to cure all the defects which could be and should be swept away. Whatever prophetical views may have been expressed before the Act came into operation, the time has now arrived when some sort of an estimate is possible as to the actual results of maternity benefit. Such an estimate has been attempted in Glasgow, where a committee was appointed some time ago to inquire into the effect of the maternity benefit of the Act on the training of the midwives of that city. The Glasgow Obstetrical and Gynæcological Society appointed the committee, whose report has now been presented. The general conclusion reached is that the comfort and security of mothers and infants have not been promoted to the extent that had been hoped for.

The committee addressed a number of queries to all the medical men practising in Glasgow, and received a large number of replies; great assistance was also obtained from several hospitals, midwifery training schools, institutes, missions, etc. The analysis which the committee provide of the evidence thus derived shows conclusively that the medical profession is not attending any larger percentage of confinements than before the Act. Whether the average midwifery fee has or has not risen as an indirect result of the Act, the report does not say; but unless this is so, the doctors are not getting any financial advantage whatever from the maternity benefit provisions of the Act. This point is of some slight importance, because one of the reiterated complaints of friendly society officials against the medical profession is that the members of the latter are "grabbing" some or all of this grant, and so "robbing" poor women of money intended to provide them with food. It is quite properly and fairly arguable that if the grant (or part of it) was to find its way into medical pockets, in return for improved or increased attendance at midwifery cases, the object of the Act would be well attained. But if, as seems to be the case in Glasgow, the medical profession is not receiving any of this money, the hollowness of the outcry against it is rendered patent to all.

The effect of the Act in regard to maternity benefit, the committee state, must be largely determined by whether or no the money set apart for the case of women in child-bed is wholly used for this purpose. They find that those who formerly made good provision for an expected confinement still do so. There is, however, a change of method: the maternity benefit is merely a substitute for thrift, and there is no evidence of a disposition to regard it as an additional provision. In other words, among even the most self-reliant of the labouring classes the maternity benefit is not improving the condition of the lying-in mothers, but is merely sapping their sense of independence and of providence. Among the less thrifty classes the money is not wholly applied on behalf of the mother, and is often diverted, mainly or entirely, to other objects altogether. This is an old story now, and there have been flagrant cases of husbands spending the whole thirty shillings on drink or for other undesirable and selfish purposes.

### Midwives' Increased Practices.

But if the doctors are not doing larger midwifery practices as a result of the Act, this is not the case with the midwives. There is no doubt that midwives in independent practice are attending very many more cases among the insured than they did formerly. It has to be remembered that the Midwives Act does not apply to Scotland. In Glasgow, say the committee, as in Scotland generally, there is no commonly recognised standard of training for maternity nurses, and no State regulation of their practice. Many who act as midwives are wholly untrained, some are inadequately trained, and only a small proportion could satisfy the requirements for registration in England. This frank confession may be gratifying to the pride of England and of our excellent English midwives, but it is little to the credit of Parliament and of the Scottish members in particular; for unquestionably if the latter demanded the extension of the Midwives Act, or some similar Act, to Scotland, the boon would not be denied.

Bearing in mind the varying degrees of proficiency among the Scottish maternity nurses, the Committee made inquiries to ascertain whether the increase of practice among these women was going mainly to those best qualified to exercise it—that is, to the better trained types. At first, apparently, there was some such tendency. Unfortunately, it seems to have been checked, and the untrained

Gamp continues in much favour. After examining into this unsatisfactory state of affairs, certain reasons which may explain, if they do not justify it, emerged. A deciding influence in the choice of a midwifery nurse is the need for someone who can do some housekeeping as well as nursing when the mother of the family is laid up. Now that there is money available, it is probable that housekeeping services which before were rendered freely by friends or neighbours have to be paid for. Thus the handy woman who will help in the housework is preferred to the midwife, or to the well-trained nurse whose whole time cannot be engaged by the poor. Crude or inefficient or negligent nursing they will put up with without a murmur, so long as the nurse will do the cooking or the housekeeping or the scrubbing. It need hardly be said that this tendency is most deleterious to the health and safety of the lying-in woman. These untrained and ignorant, if usually well-meaning, women who will do this kind of double duty are dangerous enough as nurses in the ordinary way; but with household duties to discharge as well, the likelihood of their infecting their patients with septic organisms, and thus sowing the seeds of puerperal fever, is vastly increased. The weight of opinion in Glasgow is rather against the supposition that there has been any notable increase of unskilled service; but the fact remains that the "handy woman" has not been ousted by the trained midwife as a result of maternity benefit, and shows no signs of being thus eliminated.

### The Effect upon Training Centres.

But that is not all. The outdoor ("district") practice of the Maternity Hospital is the only means in the city by which practical instruction can be given to medical students in the conduct of normal labour; and this practice is also utilised for the training of nurses and midwives. Formerly the supply of, and demand for, such cases as opportunities of training were fairly well balanced. But since the Act came in there has been a reduction in the number of "district" cases attended by the students of the Maternity Hospital amounting to some 30 per cent. of the whole. This falling off is already very seriously hampering practical training, and is thus indirectly lowering the standard of proficiency among the Glasgow midwives—to say nothing of the medical students, the practitioners of to-morrow. While it may be possible by adjustment so to utilise these restricted resources that the minimum requirements of the examining boards shall be met, such an enforced limitation is to be deplored, and it is quite possible that the present minimum may have to be lowered still further.

To the general question whether maternity benefit had favoured the comfort and safety of mother and child, fifty-seven practitioners replied in the affirmative and thirty-six in the negative. Sixteen said they had observed no change, three that the Act had been definitely prejudicial, twenty-four that the effect was doubtful or partly beneficial and partly prejudicial, and thirty would express no opinion. The committee conclude that such a want of decisiveness in the general result of the replies to this question in itself indicates that maternity benefit has not promoted in Glasgow the comfort and security of mothers and infants to the extent that might have been hoped for. Those who desire to read for themselves the findings of this committee in greater detail will find it useful to consult the *Glasgow Herald*, June 6, 1914.

### The Influence of the Act upon Maternity Charities.

One institution in particular greatly hit by the Act, as was pointed out by Mr. Edgar Kemp in a paper recently read before the Council of the Charity Organisation Society, is the Royal Maternity Charity. This organisation, which was established in 1757, has two objects. It treats expectant mothers during their confinement and at the same time fulfils the equally useful function of training midwives. A century ago 5,000 cases a year were dealt with, but this number fell until in 1912 the figure was 2,156. In 1913 the cases numbered 855, a reduction of 61 per cent.

Naturally, the Charity has for years been affected by the competition of the general and lying-in hospitals, to whom it is an important matter to find material for the training of medical students and midwives, but the heavy decrease in 1913 was attributed to the altered conditions under the National Health Act. This year, by an arrangement between the midwives and the institution, patients bearing letters of recommendation from the Charity will be treated free of charge.

Among the general hospitals the out-patients in 1913 applying for relief in respect of maternity showed a reduction of 13 per cent. as compared with the previous year. This, of course, is a serious matter to hospitals with medical schools, it being essential that the students should have instruction and practice in the delivery of children. The situation in some parts of London at the present time is one of great difficulty, as sufficient maternity cases for the proper education of the students and midwives are not forthcoming. In one particular district, where the resident population is on the decrease, there is keen competition between two general hospitals to obtain patients, and the effect no doubt will ultimately be that the present provision by the State of 30s. will be entirely retained by the patient, the hospital doing all that is necessary free of charge. In other parts of London where competition is not so keen a fixed charge to maternity patients is made, and this system is in force at most of the chief lying-in hospitals.

In summing up the whole question in his interesting paper, Mr. Kemp remarks: "Finally, the question which arises is how far can the plea, 'We must keep up our medical school,' be held to excuse the methods which some hospitals adopt to secure patients? It seems to be commonly assumed that such a plea is sufficient. Is not the proper solution, however, a better distribution of the institutions, and a better understanding as to the area that each should cover?"

